# Discovery of a new species of the genus *Triarthron* Märkel, 1840 (Coleoptera, Leiodidae) with a key to Japanese species of the tribe Sogdini

**DOI:** 10.3897/zookeys.938.51614

**Published:** 2020-06-04

**Authors:** Hideto Hoshina

**Affiliations:** 1 Faculty of Education, University of Fukui, Fukui City, 910–8507, Japan University of Fukui Fukui Japan

**Keywords:** Japan, Leiodinae, round fungus beetles, taxonomy, *Triarthron itoi* sp. nov., *Triarthron maerkelii*

## Abstract

In the genus *Triarthron* (Coleoptera, Leiodidae, Leiodinae), only two species are known to occur in Palearctic and Nearctic regions. In this paper, a new species in Japan, *Triarthron itoi* Hoshina, **sp. nov.**, is described. This brings the number of species in the genus to three. A key to the Japanese species of the tribe Sogdini is given.

## Introduction

The genus *Triarthron* belongs to the tribe Sogdini of the subfamily Leiodinae in the family Leiodidae (Newton 1998; Perreau 2004, 2015) and was established based on a European species, *T. maerkelii* by Märkel (1840). In the Palearctic region, *T. punctipennis* Reitter, 1901 was described by Reitter (1901) but transferred to the genus *Deltocnemis* J. Sahlberg, 1886 by Reitter (1913). Finally, *D. puctipennis* (Reitter) was synonymized with Stereus (Deltocnemis) hamatus J. Sahlberg, 1886 (Perkovsky 1991).

Later, Israelson (1978) added one species, *T. thurepalmi* Israelson, 1978 to a member of *Triarthron* from the Canary Islands, but this species was also transferred to the genus *Stereus* Wollaston, by Daffner (1983). It was cleared that *Stereus* Wollaston was a homonym of *Stereus* Mannerheim, 1846 (Curculionidae) (Bouchard et al. 2011), and the species was later transferred to the genus *Pseudotriarthron* Normand, 1938 by Sáez Bolaño et al. (2013).

In the Nearctic region, Horn (1868) described *Triarthron lecontei* Horn, 1868 from California. Later, Schaufuss (1882) added one Californian species, *T. cedonulli* Schaufuss, 1822, to the fauna of *Triarthron*, but Horn (1883) synonymized that species with *T. lecontei*. Furthermore, Horn (1883) described a new species, *T. pennsylvanicum* Horn, 1883 from Pennsylvania. However, that species was synonymized with *T. lecontei* Horn, 1868 by Peck and Cook (2009). Thus, *Triarthron* is a small genus, and is composed of only two species, *T. maerkelii* and *T. lecontei*, worldwide.

Hisamatsu (1985) recorded *Triarthron maerkelii* Märkel, 1840 for the first time in Japan. Recently, I had an opportunity to examine one unidentified Japanese *Triarthron* specimen. My careful examination showed that the specimen is a new member of the genus. I describe the new species under the name *Triarthron itoi* Hoshina sp. nov., as a third member of the genus.

In Japan, five species in four genera of the tribe Sogdini have been recorded: *Triarthron*, *Hydnobius* Schmidt, 1841; *Hinomoto* Hoshina, 2002; and *Sogda* Lopatin, 1961 (Hisamatsu 1985; Hoshina 2002, 2010, 2012, 2014, 2015; Hoshina and Sunada 2003). *Triarthron itoi* Hoshina, sp. nov. is a sixth Japanese species of Sogdini. Below, I provide a key to the Japanese species of Sogdini.

## Materials and methods

All specimens used in this study were deposited in the following collections:

**EUMJ** Ehime University, Matsuyama, Japan

**FU** University of Fukui, Fukui, Japan

**OSAKA** Osaka Museum of Natural History, Osaka, Japan

The methods are the same as those described in Hoshina (2012).

## Key to Japanese species of the Sogdini tribe

**Table:** 

1	Pronotum almost as long as wide	**2**
–	Pronotum clearly wider than long (Fig. 1)	**3**
2	Median lobe of the aedeagus is relatively slender and weakly curved in lateral view	***Hinomoto nihonensis* Hoshina**
–	Median lobe of the aedeagus is relatively thick and almost straight in lateral view	***Hinomoto bungensis* Hoshina**
3	Antennae forming a club on antennomeres 9–11 (Fig. 3)	**4**
–	Antennae forming a club on antennomeres 7–11	**5**
4	Body length 4.8 mm; mesofemur with relatively large teeth at dorsal lamina of posterior margin (Fig. 4); metafemur strongly expanded anteriorly at about half of antero-apical margin (Fig. 5); median lobe of aedeagus weakly and simply curved at lateral margins in dorsal view (Fig. 8)	***Triarthron itoi* Hoshina, sp. nov.**
–	Body length 2.5–3.8 mm; mesofemur with teeth less-visible because of being hidden by ventral side of mesofemur (Fig. 6); metafemur relatively weakly expanded anteriorly at about half of antero-apical margin (Fig. 7); median lobe sharply narrowed from about apical 2/5 towards apex (Fig. 10)	***Triarthron maerkelii* Märkel.**
5	Body cylindrical in general; apex of right mandible bidentate	***Hydnobius akitsuensis* Hoshina**
–	Body long oval in general; apices of both mandibles simply pointed	***Sogda hadai* Hoshina**

## Taxonomy

### Triarthron itoi

Taxon classificationAnimaliaColeopteraLeiodidae

Hoshina sp. nov.

3551D951-E432-5E6E-9E51-D6C99630554A

http://zoobank.org/198DA238-E24B-4329-A2B8-7A197EAAE909

**Figure 1.**
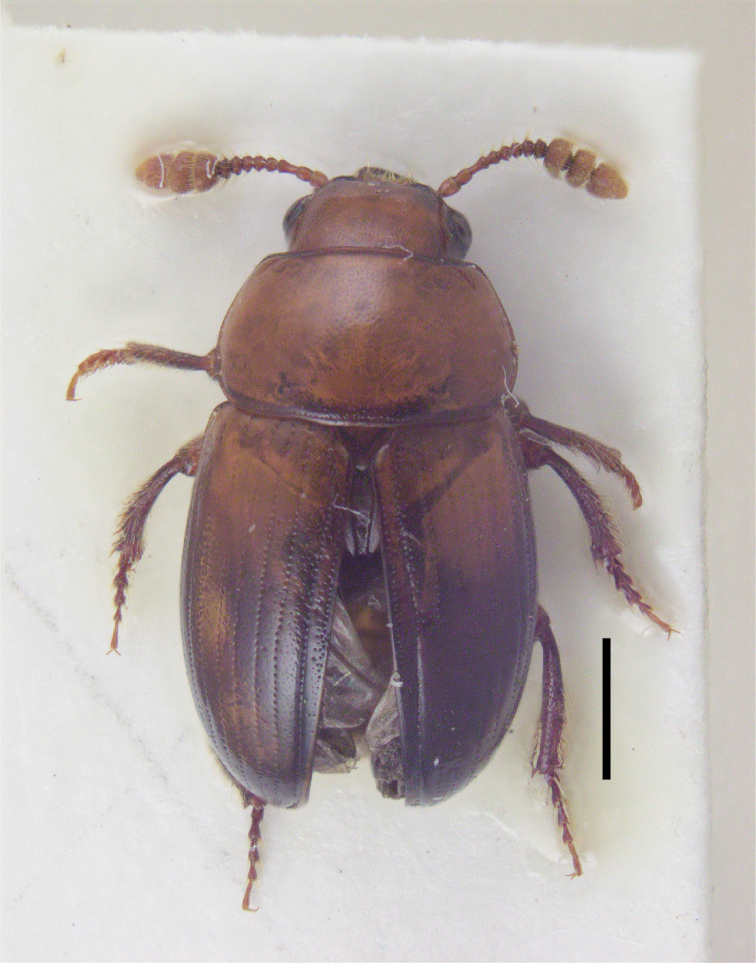
Habitat of *Triarthron itoi* Hoshina, sp. nov. Scale bar: 1 mm.

**Figures 2–7.**
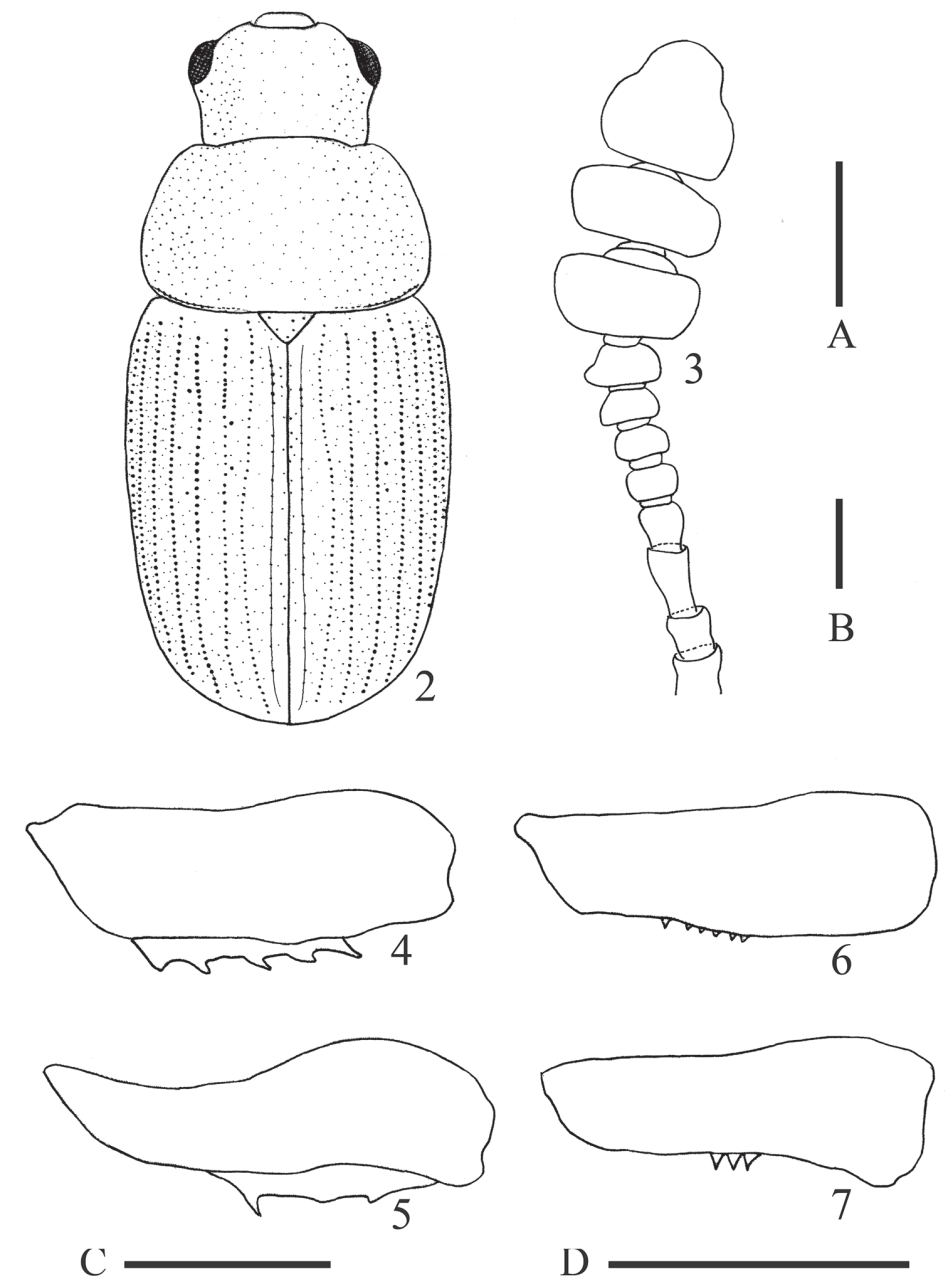
*Triarthron itoi* Hoshina, sp. nov. (**2–5**). **2** Body **3** right antennomere, dorsal view **4** left mesofemur, ventral view **5** left metafemur, ventral view. *T. maerkelii* Märkel (**6, 7)**. **6** Left mesofemur, ventral view **7** left metafemur, ventral view. Scale bar: A: 1 mm (**2**), B: 0.2 mm (**3**), C, D: 0.5 mm (**4–7**).

**Figures 8–10.**
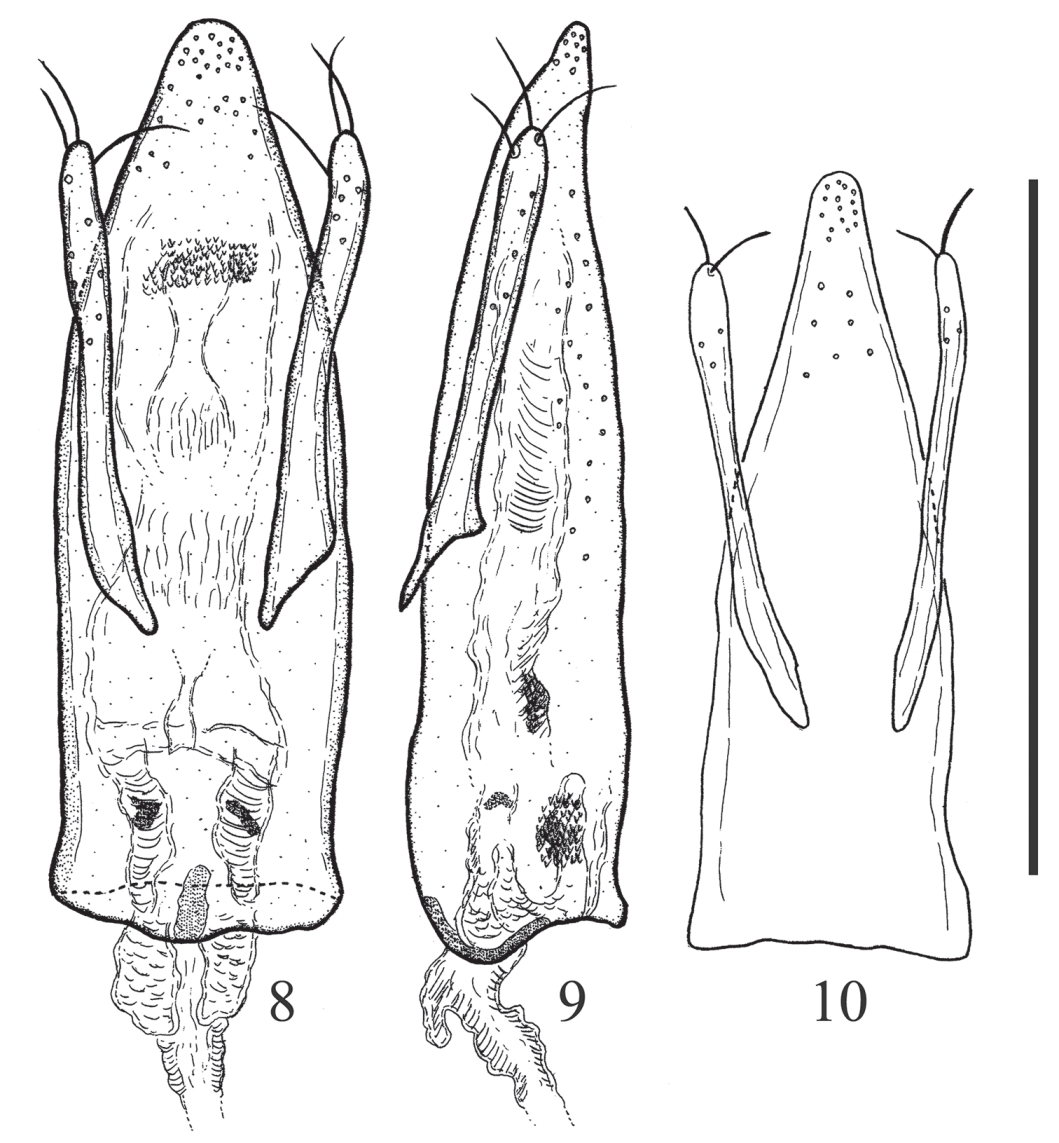
*Triarthron itoi* Hoshina sp. nov. (**8, 9**). **8** Aedeagus, dorsal view **9** ditto, lateral view. *T. maerkelii* Märkel (**10**) aedeagus, dorsal view. Scale bar: 0.5 mm (**8–10**).

Figures 1 , 2–5 , 8 , 9

#### Type locality.

Japan, Honshu: Nara Prefecture, Nara City, Nara Park, 34°41'4"N, 135°50'36"E (DMS).

#### Material examined.

***Holotype***, ♂ (OSAKA. Type No.: OMNH TI 528): Japan, Honshu, Nara Prefecture, Nara City, Nara Park, 34°41'4"N, 135°50'36"E (DMS), 16.xii.2018, F. Itô leg. (OSAKA).

#### Specimens examined of related species.

*Triarthron maerkelii* Märkel, 1840. 3 ♂♂, 1 ♀ (EUMJ), Japan – Honshu, Gunma Prefecture, Tsumagoi Village, Mt. Asashiki, 24.vii.1979, K. Itô leg.; 1 ♂ (FU), Japan – Hokkaido, Ebetsu City, Nopporo Forest Park, 29.vi.2000, S. Hori leg.; 1 ♂ (FU), Sweden, SM. Hornsö, 27.vi.1998, B. Andersson leg.

#### Diagnosis.

Body length about 5 mm. Dorsum is almost concolorous, brown. Both mandibles were sharply curved inwardly at about apical 1/4. Mesofemur bearing five small teeth at dorsal lamina of posterior margin. Metafemur strongly expanded anteriorly at about half of the antero-apical margin and bearing a relatively long tooth and two tiny teeth at the dorsal lamina of the posterior margin. The median lobe of aedeagus weakly curved at lateral margins in dorsal view.

#### Description.

***Measurement of holotype***. Body 4.8 mm in length; head 1.1 mm in length (from the front margin of the clypeus to base) and 1.3 mm in width; pronotum 1.2 mm in length and 2.0 mm in width; elytra 2.8 mm in length and 2.2 mm in width.

***Coloration.*** Dorsum shining and almost concolorous, brown (Fig. 1); clypeus and labrum light brown; antennae brown and terminal three antennomeres slightly lighter than others; legs brown in general, but all trochanters and about basal 2/5 of metafemora blackish brown; mesoventrite, metaventrite, and abdominal ventrites light brown.

***Body*** approximately 2.2 times as long as wide.

***Head*** almost smooth, minutely, and densely punctate (Fig. 2), and bearing a few short and very fine setae near each eye and a few long and fine setae near each lateral-basal corner of the clypeus; both mandibles sharply curved inwardly at about apical 1/4 and lacking large teeth at internal margins; antennomeres 1–3 longer than wide; antennomeres 4 and 11 about as long as wide; remaining antennomeres wider than long (Fig. 3).

***Pronotum*** almost smooth and glabrous, widest at about basal 2/5 of lateral margins, minutely and densely punctate as head (Fig. 2), and with a transverse fine groove along the basal margin, which is interrupted at the central part (Fig. 2).

***Scutellum*** almost smooth and distinctly punctate (Fig. 2).

***Elytra*** almost smooth and glabrous except for very sparse and fine setae along lateral margins, widest at about basal 1/3 of lateral margins (Fig. 2); each elytron bearing nine rows of punctures and ninth row present along lateral-downside margins and invisible in dorsal view (Fig. 2); punctures comprising nine rows of punctures distinct and larger than those of head and pronotum (Fig. 2); punctures between rows of punctures dense and minute (Fig. 2).

***Hind wings*** fully developed.

***Mesoventrite*** weakly microreticulate and almost glabrous; metaventrite and abdominal ventrites distinctly microreticulate, and densely and finely pubescent.

***Legs*** with many small spines as other species of the genus *Triarthron*; mesofemur approximately 2.8 times as wide as long, weakly expanded anteriorly at about half of antero-apical margin, and bearing five small teeth at dorsal lamina of posterior margin (Fig. 4); mesotibia weakly curved inwardly; metafemur approximately 2.8 times as wide as long, strongly expanded anteriorly at about half of antero-apical margin, and bearing a relatively long tooth and two tiny ones at dorsal lamina of posterior margin (Fig. 5); metatibia almost straight.

***Aedeagus*** slender in general (Figs 8, 9); median lobe weakly and simply curved at lateral margins, round at apex in dorsal view (Fig. 8) and almost straight in lateral view (Fig. 9); both parameres almost symmetrical and round at apex (Fig. 8) and almost straight in lateral view (Fig. 9); each paramere bearing three apical setae (Figs 8, 9).

#### Etymology.

The specific name is dedicated to Mr. Fukuo Itô, the collector of the holotype.

#### Distribution.

Japan: Honshu (Nara Prefecture).

#### Differential diagnosis.

Collecting the tribe Sogdini is generally not easy in Japan and identified Japanese specimens of the tribe are very small in quantity. *Triarthron itoi* Hoshina, sp. nov. is described based on only one specimen; therefore, the degree of individual variation cannot be determined in this species. However, I found some morphological features on that specimen that are clearly different from two known species of *Triarthron*, and recognize it as a new member of the genus. *Triarthron itoi* sp. nov. can be distinguished from *T. maerkelii* Märkel, 1840 by the following features: it has a large body whose length is 4.8 mm, mesofemur with relatively large teeth at the dorsal lamina of the posterior margin (Fig. 4), metafemur strongly expanded anteriorly at about half of the antero-apical margin (Fig. 5), and median lobe of aedeagus weakly and simply curved at lateral margins in dorsal view (Fig. 8). In contrast, *T. maerkelii* differs in the following ways: it has a relatively small body whose length is 2.5–3.8 mm (Daffner 1983), mesofemur with teeth less-visible because of being hidden by ventral side of mesofemur (Fig. 6), metafemur relatively weakly expanded anteriorly at about half of antero-apical margin (Fig. 7), and median lobe sharply narrowed from about apical 2/5 towards apex (Fig. 10).

Moreover, *T. itoi* sp. nov. can be separated from *T. lecontei* Horn, 1868 by having both mandibles sharply curved inwardly at about apical 1/4 and lacking large teeth at internal margins, and metafemur strongly expanded at the anterior margin (Fig. 5). In contrast, *T. lecontei* has both mandibles relatively weakly curved inwardly at internal margins, right one with an elongated sub-apical tooth, and metafemur almost straight at the anterior margin (Hatch 1957; Peck and Cook 2009).

#### Natural history.

Life history of *Triarthron itoi* Hoshina, sp. nov. is not known.

## Supplementary Material

XML Treatment for Triarthron itoi
